# Effect of plasma vitamin C levels on Parkinson’s disease and age at onset: a Mendelian randomization study

**DOI:** 10.1186/s12967-021-02892-5

**Published:** 2021-05-24

**Authors:** Haijie Liu, Yan Zhang, Haihua Zhang, Longcai Wang, Tao Wang, Zhifa Han, Liyong Wu, Guiyou Liu

**Affiliations:** 1 Department of Neurology, Xuanwu Hospital, Capital Medical University, Beijing, 100053 China; 2 Department of Pathology, The Affiliated Hospital of Weifang Medical University, Weifang, 261053 China; 3 Beijing Institute of Brain Disorders, Laboratory of Brain Disorders, Ministry of Science and Technology, Collaborative Innovation Center for Brain Disorders, Capital Medical University, Beijing, 100069 China; 4 Department of Anesthesiology, The Affiliated Hospital of Weifang Medical University, Weifang, 261053 China; 5 Academy for Advanced Interdisciplinary Studies, Peking University, Beijing, China; 6 Chinese Institute for Brain Research, Beijing, China; 7 School of Medicine, School of Pharmaceutical Sciences, THU-PKU Center for Life Sciences, Tsinghua University, Beijing, China; 8 Beijing Key Laboratory of Hypoxia Translational Medicine, Xuanwu Hospital, Capital Medical University, Beijing, 100053 China; 9 National Engineering Laboratory of Internet Medical Diagnosis and Treatment TechnologyXuanwu Hospital, Capital Medical University, Beijing, 100053 China

**Keywords:** Parkinson’s disease, Vitamin C, Genome-wide association study, Mendelian randomization, Inverse-variance weighted

## Abstract

**Background:**

Until now, epidemiological evidence regarding the association between vitamin C intake (both diet and supplements) and Parkinson’s disease (PD) remains inconsistent. Hence, it is necessary to establish the causal link between vitamin C levels and PD, and further develop effective therapies or prevention.

**Methods:**

We selected 11 newly identified plasma vitamin C genetic variants from a large-scale plasma vitamin C GWAS dataset (n = 52,018) as the effective instrumental variables, and extracted their corresponding GWAS summary statistics from PD (33,674 PD cases and 449,056 controls) and PD age at onset (AAO) (n = 28,568). We then performed a Mendelian randomization (MR) study to evaluate the causal association of plasma vitamin C levels with PD and PD AAO using inverse-variance weighted (IVW), the weighted median, MR-Egger, and MR-PRESSO test.

**Results:**

We did not observe any significant association between genetically increased vitamin C levels and PD. Interestingly, we found a reduced trend of PD AAO (1.134 years) with 1 SD genetically increased vitamin C levels using IVW (beta = − 1.134, 95% CI: [− 2.515, 0.248], *P* = 0.108). Importantly, this trend was further successfully verified using both weighted median and MR-Egger. Each 1 SD genetically increased vitamin C levels could reduce PD AAO 1.75 and 2.592 years using weighted median (beta = − 1.750, 95% CI: [− 3.396, − 0.105], *P* = 0.037) and MR-Egger (beta = − 2.592, 95% CI: [− 4.623, − 0.560], *P* = 0.012).

**Conclusions:**

We demonstrated the causal association between genetically increased plasma vitamin C levels and reduced PD AAO in people of European descent. Randomized controlled trials are required to clarify whether diet intake or supplement, or both could reduce the AAO of PD.

## Background

Parkinson’s disease (PD) is the second most common neurodegenerative disease in the elderly [1, 2]. Evidence shows that oxidative stress is involved in the degeneration of dopaminergic neurons in PD [3]. Vitamin C is a major antioxidant and a neuromodulator in dopaminergic neurons, which could neutralize reactive oxygen species and reduce oxidative stress [4, 5]. Observational study indicated significantly reduced lymphocyte vitamin C levels in patients with severe PD compared with less severe PD patients [5]. Meanwhile, a reduced trend in plasma vitamin C levels in patients with severe PD was also reported [5]. These findings show that high vitamin C intake (both diet and supplements) may be theoretically beneficial for PD treatment or prevention.

Until now, epidemiological evidence regarding the association between vitamin C intake (both diet and supplements) and PD remains inconsistent. In 1997, the community-based Rotterdam Study in the Netherlands indicated that high dietary intake of vitamin C could not decrease the risk of PD with odds ratio (OR) = 0.9 (95% confidence interval (CI): 0.4–1.9) per 100-mg vitamin C intake [6]. In 2002, the Nurses’ Health Study and the Health Professionals Follow-Up Study identified that none of the total vitamin intake, vitamin C supplement, and dietary vitamin C intake, was significantly associated with the risk of PD [7]. In 2011, a Japan multicenter hospital‐based case control study indicated that higher dietary intake of vitamin C was not associated with the decreased risk of PD [8]. In 2016, the Nurses’ Health Study and the Health Professionals Follow-up Study showed that vitamin C intake from diet could significantly reduce the risk of PD [9]. However, this significant association was not successfully replicated in a 4-year lag analysis [9]. Meanwhile, the combined vitamin C intake from diet and supplements was not associated with the PD risk [9]. In 2017, the Swedish Mammography Cohort (SMC) and the Cohort of Swedish Men (COSM) study found that dietary vitamin C intake was inversely associated with PD risk in women (HR = 0.91, 95% CI: 0.83–1.00) [10]. In 2021, the Swedish National March Cohort study (43,865 men and women aged 18–94 years with a mean follow-up time of 17.6 years) found that individuals with the highest dietary vitamin C had the reduced PD risk (hazard ratio (HR) = 0.68; 95% CI: 0.52–0.89) compared with those the lowest dietary vitamin C [11].

Hence, the causal link between vitamin C levels and PD remains unclear. In recent years, Mendelian randomization (MR) design has been widely used to determine the causal inferences and could overcome the methodological limitations of observational studies [12]. Here, we performed a MR study to investigate the causal association between plasma vitamin C levels and PD using multiple large-scale genome-wide association study (GWAS) datasets from plasma vitamin C, PD and PD age at onset (AAO) [13–15].

## Methods

### Study design

**Fig. 1:**
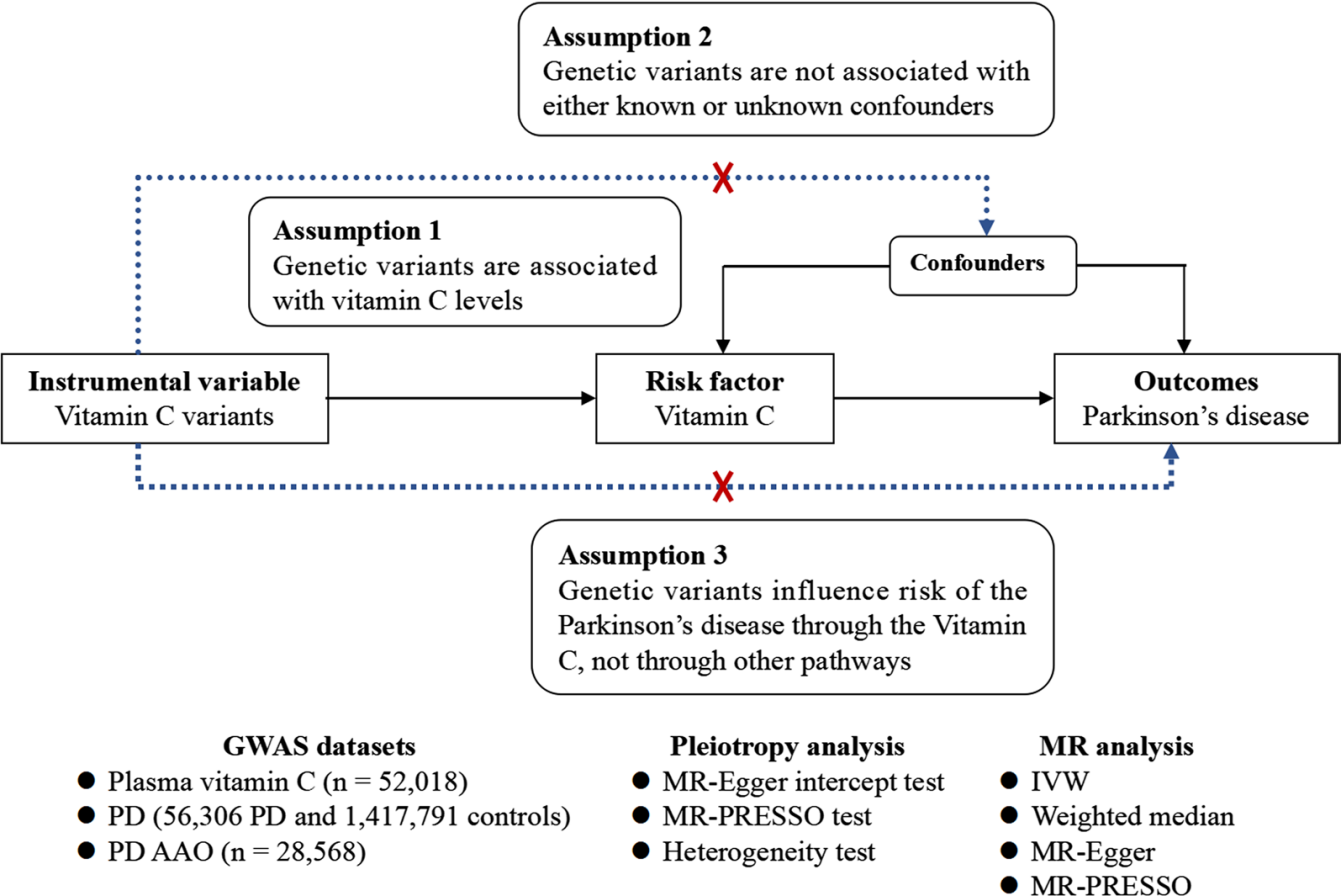
The flow chart about the MR study design

This MR study is based on the large-scale GWAS summary datasets in plasma vitamin C, PD and PD AAO [13–15]. All participants have given informed consents in all these corresponding original studies [14, 15]. In general, MR must meet three principal assumptions, as provided in Fig. 1, a flow chart about our MR study design. The second and third assumptions are collectively known as independence from pleiotropy, as described in recent studies [16–18].

### Plasma vitamin C genetic variants

Typically, independent genetic variants with genome-wide significance (*P* < 5 × 10^−8^) are selected as the potential instruments in MR studies, as described in recent studies [19–22]. Here, we selected 11 independent genetic variants with a genome-wide significant level (*P* < 5 × 10^−8^) from a recent plasma vitamin C GWAS dataset in 52,018 individuals of European ancestry [13]. This GWAS is based on a large-scale meta-analysis in four populations including the Fenland study, European Prospective Investigation into Cancer and Nutrition (EPIC)-InterAct study, EPIC-Norfolk study, and EPIC-CVD study [13]. The summary results regarding the effect of each genetic variant on vitamin C levels and the standard errors were provided in Table 1.

**Table 1:** Main characteristics of 11 selected plasma vitamin C genetic variants

SNP	Chromosome	Position (GRCh37)	EA	NEA	EAF	Beta	SE	*P* value	Gene	*R*^2^^(%)^
rs6693447	1	2330190	T	G	0.551	0.039	0.006	6.25E−10	*RER1*	0.08
rs13028225	2	220031255	T	C	0.857	0.102	0.009	2.38E−30	*SLC23A3*	0.2
rs33972313	5	138715502	C	T	0.968	0.36	0.018	4.61E−90	*SLC23A1*	0.76
rs10051765	5	176799992	C	T	0.342	0.039	0.007	3.64E−09	*RGS14*	0.06
rs7740812	6	52725787	G	A	0.594	0.038	0.006	1.88E−09	*GSTA5*	0.08
rs174547	11	61570783	C	T	0.328	0.036	0.007	3.84E−08	*FADS1*	0.05
rs117885456	12	96249111	A	G	0.087	0.078	0.012	1.70E−11	*SNRPF*	0.08
rs2559850	12	102093459	A	G	0.598	0.058	0.006	6.30E−20	*CHPT1*	0.18
rs10136000	14	105253581	A	G	0.283	0.04	0.007	1.33E−08	*AKT1*	0.06
rs56738967	16	79740541	C	G	0.321	0.041	0.007	7.62E−10	*MAF*	0.07
rs9895661	17	59456589	T	C	0.817	0.063	0.008	1.05E−14	*BCAS3*	0.12

Beta is the regression coefficient based on the vitamin C raising allele (effect allele); R^2^, the proportion of vitamin C variance explained by the selected genetic variants

*SNP* single-nucleotide polymorphism, *EA* effect allele, *NEA* non-effect allele, *EAF* effect allele frequency, *SE* standard error

### PD GWAS dataset

The PD GWAS dataset is from the International Parkinson’s Disease Genomics Consortium (IPDGC) that conducted the large-scale meta-analysis of 17 GWAS datasets in 56,306 PD cases (37,688 PD cases, 18,618 UK Biobank proxy-cases) and 1,417,791 control individuals of European ancestry [14]. However, the GWAS summary statistics from the meta-analysis of all these selected 17 GWAS datasets are not publicly available. Hence, we selected the subgroup of these 17 GWAS datasets including 14 GWAS datasets by excluding Nalls and colleagues, 23 and Me post-Chang and colleagues and Web-Based Study of Parkinson’s disease [14]. The subgroup GWAS dataset included 33,674 PD cases and 449,056 controls [14]. Table 2 provides the demographic profiles about the 14 PD GWAS datasets, as provided in the original study [14].

**Table 2:** Demographic profiles about the PD GWAS dataset

Study	Cases	Controls	Female cases (%)	Female control (%)	Case age at onset (mean, SD)	Control age at last exam (mean, SD)
Baylor College of Medicine/University of Maryland	769	195	33.81	69.74	64.83 (10.12)	65.48 (8.31)
Finnish Parkinson’s	386	493	45.85	78.9	55.27 (5.64)	92.35 (3.86)
Harvard Biomarker Study (HBS)	527	472	34.35	61.65	66.31 (10.07)	69.9 (9.02)
McGill Parkinson’s	582	905	34.54	48.4	65.71 (9.79)	55.79 (10.69)
Oslo Parkinson’s Disease Study	476	462	35.71	42.21	65.32 (9.28)	61.85 (11.06)
Parkinson’s Disease Biomarker’s Program (PDBP)	512	282	38.67	51.06	64.46 (9.37)	62.19 (10.73)
Parkinson’s Progression Markers Initiative (PPMI)	363	165	33.06	33.33	64.24 (9.65)	63.79 (10.59)
System Genomics of Parkinson’s Disease (SGPD)	1169	968	35.24	53.93	59.88 (10.86)	66.64 (9.65)
Spanish Parkinson’s (IPDGC)	2110	1333	43.13	54.39	63.92 (12.54)	64.03 (12.59)
Tubingen Parkinson’s disease cohort (CouragePD)	666	542	36.04	57.93	59.89 (11.25)	67.48 (8.41)
Vance (dbGap phs000394)	620	299	27.74	50.84	77.47 (8.40)	81.98 (12.78)
UKPDMED (CouragePD)	1025	655	32.78	72.67	NA	NA
UKBioBank	18,618	436,419	57.62	54.14	58.45 (7.20)	56.69 (8.05)
NeuroX—dbGaP (phs000918.v1.p1)	5851	5866	NA	NA	NA	NA
All	33,674	449,056	NA	NA	NA	NA

### PD AAO GWAS dataset

The PD AAO GWAS dataset is from the large-scale meta-analysis of 18 PD AAO GWAS datasets in 28,568 PD cases including 17 independent cohorts from IPDGC (n = 17,996) and the 23andMe PD cohort (n = 10,572) [15]. The average AAO in the IPDGC dataset was 62.14 (range 20–96, SD = 12.08), and average AAO in the 23andMe dataset was 60.71 (range 40–97, SD = 9.98) [15]. Table 3 provides the demographic profiles about the 18 PD AAO GWAS datasets, as provided in the original study [15].

**Table 3:** Demographic profiles about the PD AAO GWAS dataset

Dataset	PD cases	Average age of onset of cases (range)	Sex ratio male/female of cases
Dutch GWAS [23]	764	54.94 (21–84)	1.74
Finnish GWAS	377	55.27 (30–66)	1.19
German GWAS [24]	663	55.84 (28–86)	1.55
Harvard Biomarker Study (HBS)	525	66.31 (35–89)	1.92
McGill Parkinson’s	580	65.56 (37–91)	1.89
IPDGC NeuroX [25]	5428	61.27 (20–89)	1.82
NIA PD GWAS [24]	845	58.25 (20–87)	1.46
OsloParkinson’s Disease Study	476	55.70 (24–83)	1.8
Parkinson’s Disease Biomarker’s Program (PDBP)	512	64.46 (34–87)	1.59
Parkinson’s Progression Markers Initiative (PPMI)	360	64.24 (36–87)	2.03
PROBAND	1815	66.25 (29–90)	1.85
PROPARK	235	55.69 (29–81)	2.09
Baylor College of Medicine/University of Maryland	764	64.83 (23–92)	1.95
Spanish GWAS [26]	1928	63.90 (20–95)	1.35
Tuebingen Parkinson’s Disease cohort	666	59.89 (23–87)	1.78
WTCCC PD GWAS [27]	1477	64.10 (23–96)	1.6
System Genomics of Parkinson’s disease (SGPD)	581	59.96 (24–84)	1.75
Total IPDGC	17,996	62.14 (20–96)	1.7
23 and Me	10,572	60.71 (40–97)	1.54
Total	28,568	61.71 (20–97)	1.64

### Pleiotropy analysis

The pleiotropy analysis is based on three different statistical methods including MR-Egger intercept test [28], MR pleiotropy residual sum and outlier (MR-PRESSO) global test [28], and heterogeneity test using Cochran’s Q statistic and $I^{2}$ statistic [29, 30]. The significance threshold is *P* < 0.05. All the statistical tests were completed using three R Packages including ‘meta: General Package for Meta-Analysis’, ‘MendelianRandomization’ and ‘MR-PRESSO’, respectively [12].

### MR analysis

We selected four MR analysis methods including the inverse-variance weighted (IVW), the weighted median, MR-Egger, and MR-PRESSO test [12, 28, 31]. The effect size (beta) and 95% confidence interval (CI) correspond to 1 standard deviation (SD) in vitamin C levels. All the statistical tests were completed using R Packages ‘MendelianRandomization’ and ‘MR-PRESSO’, respectively [12]. The significance threshold is *P* < 0.05.

### Power analysis

The proportion of vitamin C variance explained by the selected genetic variants R^2^.

$$R^{2} = \sum\limits_{i = 1}^{K} {\frac{{\beta_{i}^{2} }}{{\beta_{i}^{2} + 2*N*se(\beta_{i} )^{2} }}}$$

Here, $\beta_{i}$ is the effect size for $SNP_{i}$, $se(\beta_{i} )$ is the standard error for $SNP_{i}$, *N* is the sample size for $SNP_{i}$, and *K* is the number of the selected genetic variants [32]. The statistical power is calculated using the web-based tool mRnd and a two-sided type-I error rate α of 0.05 [33].

## Results

### Vitamin C genetic variants with PD and PD AAO

We successfully extracted the summary statistics corresponding to the 11 vitamin C genetic variants in PD and PD AAO GWAS datasets, respectively. It is noted that rs56738967 (C/G, C with the minor allele frequency (MAF) = 0.321) is an ambiguous palindromic variant (i.e. with alleles either A/T or C/G). Hence, we selected the allele frequency to distinguish the effect allele in both GWAS datasets. More detailed information about the association of these 11 vitamin C genetic variants with PD and PD AAO is proved in Table 4.

**Table 4:** Association of 11 vitamin C genetic variants in PD and PD AAO

SNP	Plasma vitamin C GWAS			PD GWAS			PD AAO GWAS		
	EA	NEA	EAF	Beta	SE	*P* value	Beta	SE	*P* value
rs10051765	C	T	0.342	0.013	0.019	0.477	0.104	0.125	0.405
rs10136000	A	G	0.283	0.003	0.021	0.872	0.100	0.141	0.478
rs117885456	A	G	0.087	− 0.053	0.043	0.216	0.274	0.267	0.305
rs13028225	T	C	0.857	0.055	0.025	0.024	− 0.325	0.167	0.051
rs174547	C	T	0.328	0.003	0.018	0.853	0.141	0.121	0.243
rs2559850	A	G	0.598	− 0.026	0.023	0.247	0.013	0.141	0.929
rs33972313	C	T	0.968	− 0.006	0.052	0.903	− 0.751	0.353	0.033
rs56738967	C	G	0.321	− 0.034	0.019	0.063	− 0.003	0.125	0.980
rs6693447	T	G	0.551	− 0.034	0.019	0.066	− 0.049	0.125	0.697
rs7740812	G	A	0.594	− 0.034	0.023	0.135	− 0.238	0.143	0.096
rs9895661	T	C	0.817	− 0.002	0.023	0.931	0.032	0.151	0.833

Beta is the regression coefficient based on the vitamin C raising allele (effect allele)

*SNP* single-nucleotide polymorphism, *EA* effect allele, *NEA* non-effect allele, *EAF* effect allele frequency, *SE* standard error

### Pleiotropy analysis

We did not identify any significant pleiotropic variant among the selected 11 vitamin C genetic variants in both the PD and PD AAO GWAS datasets using the three statistical methods with all *P* values > 0.05. More detailed pleiotropy analysis results are provided in Table 5. Hence, all these selected 11 vitamin C genetic variants could be taken as the effective instrumental variables in MR analysis.

**Table 5:** Pleiotropy analysis of 11 selected plasma vitamin C genetic variants

GWAS dataset	MR-Egger intercept			MR-PRESSO	Heterogeneity test		
	intercept	95% CI	*P* value	*P* value	*I*^2^ (%)	95% CI	*Q* *P* value
PD	− 0.016	[− 0.043, 0.011]	0.243	0.08	42.4	[0.0%; 71.5%]	0.0669
PD AAO	0.127	[− 0.011, 0.266]	0.072	0.271	15.6	[0.0%; 56.1%]	0.2951

The significance threshold is *P* < 0.05

### MR analysis

**Fig. 2:**
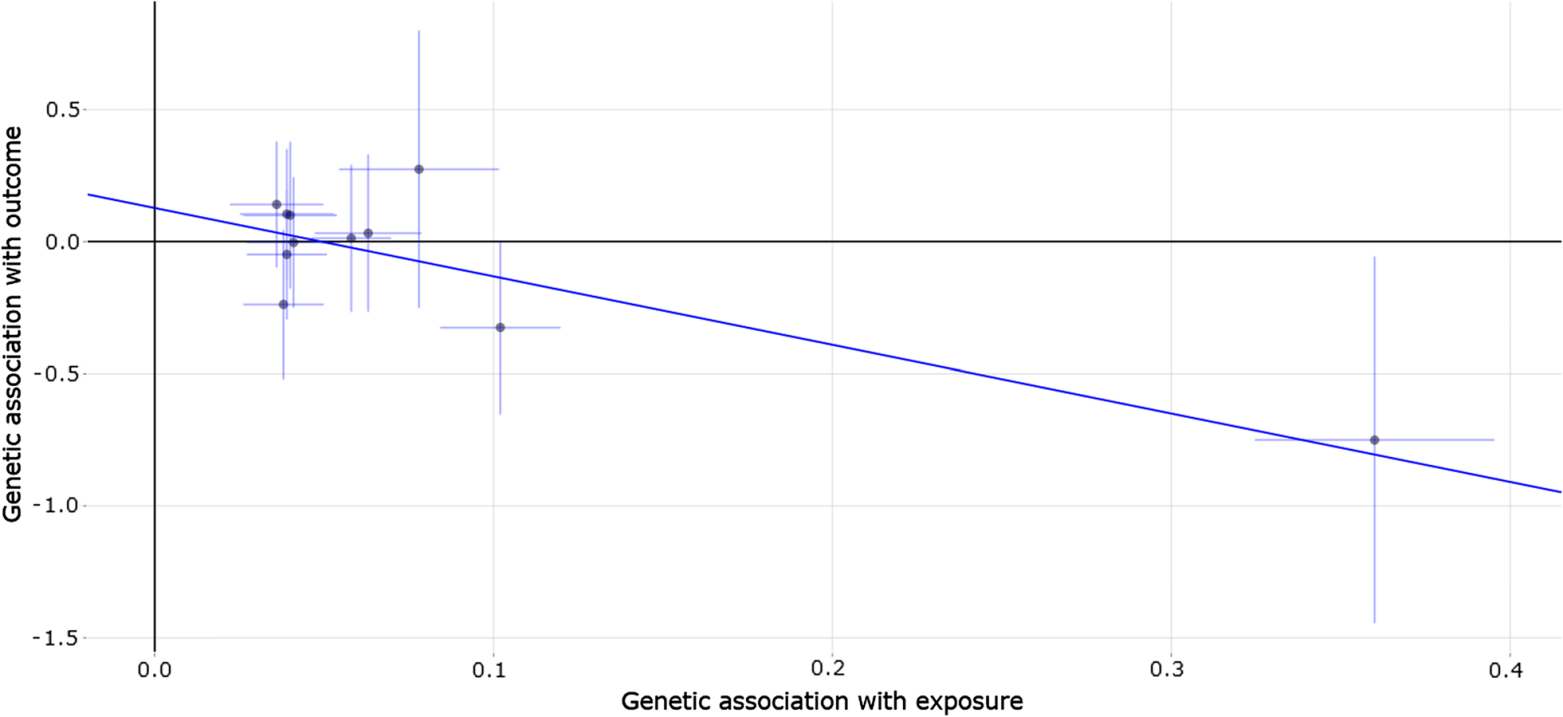
Individual estimates about the causal effect of plasma vitamin C levels on PD AAO using MR-Egger method. The x-axis shows the single nucleotide polymorphism (SNP) effect, and standard error, on plasma vitamin C levels for each of the 11 SNPs, and the y-axis shows the SNP effect, and standard error on PD AAO. The regression line for the MR-Egger method is shown

In PD GWAS dataset, we did not observe any significant association between genetically increased vitamin C levels and PD risk using all the four selected MR methods, as described in Table 6. Interestingly, we found a reduced trend of PD AAO (1.134 years) with 1 SD genetically increased vitamin C levels using IVW (beta = − 1.134, 95% CI: [− 2.515, 0.248], *P* = 0.108). Importantly, this reduced trend was further successfully verified using both weighted median and MR-Egger. In brief, each 1 SD genetically increased vitamin C levels could reduce PD AAO 1.75 and 2.592 years using weighted median (beta = − 1.750, 95% CI: [− 3.396, − 0.105], *P* = 0.037) and MR-Egger (beta = − 2.592, 95% CI: [− 4.623, − 0.560], *P* = 0.012). These estimates were consistent in terms of direction and magnitude, as provided in Table 6. Figure 2 shows the individual MR estimates about the causal effect of vitamin C levels on PD AAO using MR-Egger method.

**Table 6:** The causal association of plasma vitamin C levels with PD and PD AAO

GWAS dataset	Method	Beta	95% CI	*P* value
PD	IVW	− 0.048	[− 0.296, 0.201]	0.708
	Weighted median	− 0.018	[− 0.272, 0.237]	0.893
	MR-Egger	0.130	[− 0.255, 0.516]	0.508
	MR-PRESSO	− 0.048	[− 0.296, 0.201]	0.716
PD AAO	IVW	− 1.134	[− 2.515, 0.248]	0.108
	Weighted median	− 1.750	[− 3.396, − 0.105]	**0.037**
	MR-Egger	− 2.592	[− 4.623, − 0.560]	**0.012**
	MR-PRESSO	− 1.134	[− 2.515, 0.248]	0.139

The significance of association between vitamin C levels and AD was at *P* < 0.05; The significant *P* values 0.037 and 0.012 were bold

*CI* confidence interval, *IVW* inverse-variance weighted, *MR-PRESSO* Mendelian randomization pleiotropy residual sum and outlier

### Power analysis

All these selected 11 genetic variants could explain 1.79% variance of plasma vitamin C levels. Our MR study had 80% power to detect an OR of 0.91 or lower per SD increase in vitamin C levels for PD. In order to calculate the power about the causal association between vitamin C levels and PD AAO, the regression coefficients from a observational study to evaluate the association between plasma vitamin C levels and PD AAO are needed including both the without confounder-adjustment and with confounder-adjustment [33]. However, this kind of observational study is not publicly available until now. Hence, we could not evaluate the power about the MR analysis in PD AAO.

## Discussion

Until now, epidemiological evidence about the association between vitamin C intake and PD remains inconsistent [6–11]. Hence, it is necessary to establish the causal link between vitamin C levels and PD, and to develop effective therapies or prevention. Hence, we selected 11 vitamin C genetic variants as the effective instrumental variables and extracted their corresponding summary statistics in large-scale PD GWAS and PD AAO datasets, respectively. We then performed a MR study to evaluate the causal association of vitamin C levels with PD and PD AAO. We found no causal association between genetically increased vitamin C levels and PD risk. Interestingly, we found that genetically increased vitamin C levels were significantly associated with reduced PD AAO. Each 1 SD genetically increased vitamin C levels could reduce PD AAO 1.134, 1.75 and 2.592 years using IVW, weighted median and MR-Egger methods, respectively.

It is noted that our findings only reflect the effect of plasma vitamin C levels on PD and its age at onset, but not the serum vitamin C levels. Until now, two observational studies have been performed to evaluate the association of serum vitamin C levels with the AAO, duration and progression of PD [34, 35]. Fernandez-Calle and colleagues compared the serum vitamin C levels using 63 PD patients using their spouses as the control group [35]. The serum levels of vitamin C did not show significant difference in between PD and controls [35]. Meanwhile, they found no correlation of serum vitamin C levels with the AAO, duration and progression (scores of the Unified PD Rating Scale or the Hoehn and Yahr staging) of PD [35]. King and colleagues measured the serum vitamin C levels in 27 PD patients and 16 age-matched control subjects [34]. They found significantly increased serum vitamin C levels in PD cases than the controls [34]. Meanwhile, there was no correlation of serum vitamin C levels with the duration or progression of PD [34].

Until now, there is not publicly available observational evidence that high plasma vitamin C levels could reduce the PD AAO. However, there is at least one study that had evaluated the association of plasma and lymphocyte C levels with the progression of PD (determined by the Hoehn-Yahr scale) using 62 PD cases [5]. The results indicated that plasma vitamin C levels tended to be lower in severe PD patients compared with those at less severe stages (OR, 0.98; 95% CI 0.96–1.00; *P* = 0.09) [34]. Meanwhile, the lymphocyte vitamin C levels were significantly lower in severe PD patients (OR = 0.87, 95% CI 0.80–0.97; *P* < 0.01) compared with those at less severe stages [5].

It is known that vitamin C contributes to many health benefits especially antioxidant properties [36, 37]. The US recommended dosage of vitamin C is 100–120 mg/day for adults [36]. However, vitamin C is also a pro-oxidative factor [37, 38]. Vitamin C could be readily oxidized, which further causes DNA damage and produce oxidative stress [37, 38]. In order to translate these genetic findings into clinical and public health implications, randomized controlled trials are required to assess the effect of plasma vitamin C levels on PD AAO, and further clarify whether diet intake or supplement, or both could reduce the AAO of PD.

Our MR study may have several strengths. First, this MR design was based on the large-scale plasma vitamin C GWAS dataset (n = 52,018) and large-scale PD GWAS dataset (33,674 PD cases and 449,056 controls) and PD AAO GWAS dataset (n = 28,568). Importantly, the individuals from all these three GWAS datasets are of European ancestry, which contribute to reduce the influence of population stratification. Second, we selected 11 independent genetic variants as the potential instruments, and further demonstrated all these selected 11 genetic variants to be the effective instrumental variables using three independent statistical methods. Third, we selected four MR methods including IVW, weighted median, MR-Egger, and MR-PRESSO. Importantly, all these four MR methods produce consistent estimates.

## Limitations

Our MR study may also have some limitations. First, our MR analysis just reflects the findings in European ancestry. The causal association between vitamin C levels and AD risk may be different across different ancestries. Hence, our findings should be further replicated in other ancestries. Second, the GWAS dataset from IPDGC is based on the clinically diagnosed PD and self-report PD-by-proxy, respectively. Hence, there may be some differences across the different diagnostic criteria. Hence, PD GWAS dataset based the clinically diagnosed criteria should further verify our findings. Third, we demonstrated that the increased plasma vitamin C levels could reduce the PD AAO. However, it remains unclear about the potential mechanisms underlying this causal association, which deserves to be thoroughly evaluated. Fourth, it is known that the sodium-dependent vitamin C transporters of the SLC23 family genes including *SLC23A1* and *SLC23A2* are involved in direct transport and regulation of vitamin C concentrations [13]. Fortunately, genetic variants associated with plasma vitamin C levels of genome-wide significance (P < 5 × 10^−8^) at *SLC23A1* (rs33972313, the strongest signal) and *SLC23A3* (rs13028225, the second strongest signal) were successfully reported in the original GWAS dataset, as provided in Table 1 [13]. However, the original GWAS did not identify any genetic variant with genome-wide significance at *SLC23A2* [13]. Hence, we could not select the genetic variants at *SLC23A2* as the potential instrumental variables using the genome-wide significance criteria. One possible reason is that SVCT2 encoded by *SLC23A2* mainly regulates tissue levels of vitamin C, which further causes its impact on circulating vitamin C to be minimal [13, 39]. The other is that the current sample size from the GWAS (n = 52,018) is not enough. Fifth, the genetically increased plasma vitamin C level does not necessarily reflect in the plasma, as the equivocal results were reported in serum vitamin C and its association with PD [34, 35]. Hence, our findings deserve further investigation.

## Conclusions

In summary, our MR analysis demonstrated the causal association between genetically increased plasma vitamin C levels and reduced PD AAO in people of European descent. Hence, maintaining adequate plasma vitamin C levels may contribute to reduce the AAO of PD. Meanwhile, additional studies are also required to further verify our findings.

## Data Availability

All relevant data are within the paper. The authors confirm that all data underlying the findings are either fully available without restriction through consortia websites, or may be made available from consortia upon request.

## Abbreviations

PD: Parkinson’s disease
MR: Mendelian randomization
GWAS: Genome-wide association study
IGAP: International Genomics of Alzheimer’s Project
MR-PRESSO: MR pleiotropy residual sum and outlier
IVW: Inverse-variance weighted
